# Metagenomic and metabolomic analyses of rumen fiber digestion in Mongolian cattle fed fresh grass versus hay

**DOI:** 10.1128/spectrum.03051-25

**Published:** 2026-01-08

**Authors:** Junzhao Xu, Jianfei Ma, Huiling Lin, Sumei Yan, Huaxin Niu

**Affiliations:** 1 College of Animal Science and Technology, Inner Mongolia Minzu University 71129 , Tongliao, China; 2 College of Animal Science, Key Laboratory of Animal Nutrition and Feed Science at Universities of Inner Mongolia Autonomous Region, Inner Mongolia Agricultural University 117454 , Hohhot, China; Tongji University, Shanghai, China

**Keywords:** Mongolian cattle, rumen microbiome, carbohydrate-active enzymes, rumen function, metabolites

## Abstract

Mongolian cattle exhibit exceptional roughage tolerance due to their rumen microbiome’s robust fiber-degrading capacity, enabling efficient utilization of low-quality forage under the Mongolian Plateau’s seasonal fluctuations. This study compared rumen microbial composition, CAZyme profiles, fermentation parameters, and metabolic pathways in cattle fed fresh grass (FG) versus hay to elucidate microbe-metabolite interactions underlying fiber digestion. Thirty non-pregnant female Mongolian cattle (460 ± 35 kg, 3–4 years old) were randomly divided into two groups (*n* = 15/group): one grazed on FG, the other housed and fed autumn-harvested hay (HG). Six animals per group were subsampled for rumen fluid collection and multi-omics analyses (*n* = 6/group, total *n* = 12). Compared with the FG group, the HG group showed an increased molar proportion of acetate and a higher acetate-to-propionate ratio, along with reduced molar proportions of propionate and butyrate in rumen fermentation parameters. Metagenomic analysis revealed a higher abundance of Bacteroidales bacteria and anaerobic fungi (including *Neocallimastix *sp. JGI-2020a and *Piromyces *sp. E2) in the HG group. Functional annotation further indicated enriched carbohydrate metabolism pathways in the HG group, along with a greater diversity of CAZymes, particularly those involved in hemicellulose and pectin degradation. Metabolomics identified 13 differentially abundant carbohydrate metabolites, with gluconolactone upregulated in the HG group. Additionally, carbohydrate metabolism pathways identified in the metabolome corroborated the reliability of the metagenomic functional annotations. Correlation network analysis revealed positive associations of Bacteroidaceae bacteria, *Neocallimastix *sp. JGI-2020a, and *Piromyces *sp. E2 with acetate, hemicellulose-degrading GHs, and carbohydrate metabolic pathways. In conclusion, hay feeding enhanced ruminal fiber degradation in Mongolian cattle through increased Bacteroidales and anaerobic fungi, diversified CAZymes (especially hemicellulases/pectinases), and upregulated carbohydrate metabolism, reflecting microbial adaptation to low-quality forage.

**IMPORTANCE:**

Mongolian cattle’s superior roughage tolerance depends on a specialized rumen microbiome that degrades fibrous substrates via diverse CAZymes. However, microbe-metabolite interactions driving fiber digestion in this breed remain poorly understood. This study revealed an increased abundance of bacteria and fungi involved in rumen fiber degradation, which may be responsible for secreting enzymes associated with hemicellulose and pectin breakdown. Furthermore, the upregulation of key metabolites, including gluconolactone, indirectly promotes acetate production through pathways such as glycolysis and the pentose phosphate pathway. These findings reveal microbial adaptations enhancing low-quality forage utilization, offering new strategies for improving ruminant efficiency in seasonal or resource-limited grazing systems.

## INTRODUCTION

The Xilingol Grassland, a quintessential temperate continental grassland in northern China, is critical for regional livestock production and ecosystem stability. This ecosystem is shaped by a harsh climate characterized by a mean annual temperature of ~3°C, low annual precipitation (~290 mm), and an extended cold season, resulting in pronounced seasonal fluctuations in forage availability and quality (1). During the growing season (June–September), herbage exhibits high crude protein (CP) content (10–20%), sufficient to meet livestock nutritional requirements. Conversely, the cold season (winter-spring) features a sharp decline in CP (5–8%) and an increase in neutral detergent fiber (NDF; 60–70%), significantly reducing forage quality and constraining animal performance (2–4). Accordingly, timely harvesting and processing of natural forage in autumn are essential to maximize its nutritional value and ensure livestock survival during the feed-deficient winter-spring season. As forage nutritive value varies dynamically with plant maturity, fresh herbage provides high nutritional benefits suited to immediate grazing needs, whereas hay offers stable feed to support livestock during periods of forage scarcity. Understanding how cattle and sheep utilize these two forage forms is thus critical for optimizing livestock production in this grassland ecosystem.

Mongolian cattle, a native breed of the Xilingol Grassland, are indispensable to local herders, providing milk, meat, and draft power while serving as a cornerstone of regional livelihoods and economic stability (5). Under traditional grazing practices, natural forage constitutes the sole feed source for these cattle. In response to seasonal fluctuations in forage availability and nutrient composition, Mongolian cattle have evolved unique rumen microbial communities with highly efficient fiber-degrading capabilities, enabling sustained growth and productivity under extreme cold, prolonged dry seasons, and nutrient scarcity (5–7).

The rumen, often termed a natural “fermentation vat,” harbors a diverse microbial community comprising bacteria, archaea, fungi, and protozoa that collaboratively degrade plant cellulose into soluble sugars. These sugars are subsequently fermented into volatile fatty acids (VFAs), microbial proteins, and other nutrients critical for the host’s energy and growth (8, 9). Instead of directly degrading cellulose, the rumen microbial community produces a diverse array of CAZymes that drive fiber degradation (10). Studies by Hagen et al. (11) and He et al. (12) indicate that bacteria primarily contribute to hemicellulose degradation, whereas anaerobic fungi specialize in lignocellulose breakdown, with GH48 and GH5 family enzymes being most prevalent. Furthermore, rumen microbiota composition and enzymatic activity are influenced by ruminant species, grazing season, diet composition, and feed additives. Research on other ruminants, such as yaks and heifers, demonstrates that fiber-rich diets promote enrichment of fiber-degrading bacteria, particularly in the phyla Bacteroidota and Firmicutes, thereby enhancing fiber degradation and VFA production (13–15).

Despite the recognized role of rumen microbiota in fiber digestion, research on specific mechanisms of fiber degradation in Mongolian cattle remains limited—particularly the core bacterial and fungal taxa, key CAZymes, and metabolic pathways converting recalcitrant plant biomass into VFAs. The advent of high-throughput multi-omics technologies, such as metagenomics and metabolomics, now enables the systematic exploration of these complex microbial-host interactions (16, 17). This study hypothesizes that: (i) hay-fed Mongolian cattle exhibit increased abundance of fiber-degrading bacteria, enhancing CAZyme secretion for cellulose degradation; and (ii) they activate additional carbohydrate metabolic pathways, increasing VFA synthesis to meet energy demands. To test these hypotheses, we compared rumen microbial composition, CAZyme profiles, fermentation parameters, and metabolic pathways in Mongolian cattle fed fresh grass (FG) versus hay to elucidate microbe-metabolite interactions driving rumen fiber digestion.

## MATERIALS AND METHODS

### Animal management and sample collection

This study was conducted at a Mongolian cattle breeding conservation base in Ulagai, Xilingol League, Inner Mongolia Autonomous Region. In July 2023, 30 female Mongolian cattle (460 ± 35 kg, 3–4 years old, non-pregnant) were randomly selected from a grazing herd on the Xilingol Grassland. The cattle were randomly allocated to two groups (*n* = 15/group). One group (FG) grazed naturally on FG dominated by *Stipa* spp., *Ulmus* spp., and *Festuca* spp., while the other (HG) was housed in the same fenced enclosure and fed hay harvested in the previous autumn. No supplemental feed was provided to either group, and all animals had *ad libitum* access to feed and water. The trial consisted of a 15-day adaptation period followed by a 45-day experimental period.

On days 43–45 of the experimental period, daily forage samples were collected from both groups using the four-point sampling method. Samples from the 3 days were thoroughly mixed and pooled to determine dietary nutrient composition (Table 1). On day 45, six cattle were randomly selected from each group (*n* = 6/group, total *n* = 12). Approximately 200 mL of rumen fluid was collected from each animal before morning feeding (~12 h after the evening feeding) using a stomach tube sampler (GCYQ-1-A, Wuhan Keli Bo Equipment Co., Ltd.). This timing was selected to align with the nadir of the diurnal cycle, minimizing variability from postprandial fermentation peaks. The initial 100 mL of rumen fluid was discarded to reduce saliva contamination. The remaining fluid was filtered through four layers of sterile gauze, aliquoted into cryogenic vials, snap-frozen in liquid nitrogen, and stored at −80°C until analysis.

**TABLE 1:** Chemical composition of fresh grass and hay^*a*^

Items	HG	FG
DM (%FM)	87.35	27.66
CP (%DM)	8.06	11.97
NDF (%DM)	71.94	54.14
ADF (%DM)	52.44	30.79

^*a*^ FM, fresh material.

### Forage nutritional analysis and rumen fermentation profile

A 200 g forage sample was dried at 65°C to constant weight to determine dry matter (DM) content. The dried sample was ground to pass through a 1 mm sieve using a cutting mill (ZM200, Retsch GmbH, Beijing, China) for nutrient analysis. CP content was measured using the Kjeldahl method (18). NDF and acid detergent fiber (ADF) contents were determined using AOAC methods (18).

For VFA analysis, rumen fluid samples were thawed at 4°C, and 0.25 mL of 25% (wt/vol) metaphosphoric acid was added to 1 mL of rumen fluid. The mixture was centrifuged at 20,000 × *g* for 15 min at 4°C. The supernatant was analyzed for VFA concentrations using a gas chromatograph (TP-2060T, Beijing Beifen Tianpu Instrument Technology Co., Ltd., Beijing, China), as described by Xu et al. (19). Analytical conditions were as follows: a 2 m × 6 mm glass column packed with Chromosorb W AW-DMCS coated with 10% FFAP; high-purity N_2_ as carrier gas at 30 mL/min; temperature program: 100°C (held 2 min), increased to 180°C at 8°C/min (held 5 min); injection port at 220°C; injection volume 1 μL; FID detector at 250°C. The fuel gas was H_2_ at 30 mL/min, and the oxidizing gas was air at 300 mL/min. Quantification was performed using external standards with peak area integration.

### Metagenomic profiling of rumen microbial

Total genomic DNA was extracted from rumen fluid samples using the E.Z.N.A. Soil DNA Kit (Omega Bio-tek, Norcross, GA, USA) following the manufacturer’s protocol. DNA concentration and purity were assessed using a TBS-380 fluorometer and a NanoDrop 2000 spectrophotometer, respectively. DNA integrity was evaluated by 1% agarose gel electrophoresis. High-quality DNA was sheared to ~400 bp using a Covaris M220 ultrasonicator (Gene Company Limited, China) for paired-end library preparation. Sequencing libraries were constructed using the NEXTFLEX Rapid DNA-Seq Kit (Bioo Scientific, Austin, TX, USA) according to the manufacturer’s instructions. Paired-end sequencing (2 × 150 bp) was performed on the Illumina NovaSeq 6000 platform (Illumina Inc., San Diego, CA, USA) using NovaSeq 6000 S4 Reagent Kits at Majorbio Bio-Pharm Technology Co., Ltd. (Shanghai, China).

Raw sequencing reads were subjected to quality filtering and trimming to remove low-quality bases and adapters. Filtered reads were aligned to the Bos taurus reference genome using BWA (v0.7.9a) (20) to remove host-derived sequences. High-quality non-host reads were assembled into contigs using MEGAHIT (v1.1.2) (21). Contigs ≥300 bp were used for open reading frame prediction with MetaGene (22). A non-redundant gene catalog was constructed using CD-HIT (v4.8.1) at 95% identity (23). Clean reads were mapped to the catalog at ≥95% identity using SOAPaligner (v2.21) (24) to generate gene abundance profiles (expressed as TPM). Representative sequences were taxonomically annotated against the NCBI NR database (accessed 4 June 2020) using DIAMOND (v0.8.35) (25). Functional annotations were performed against the KEGG database (v94.2), and CAZymes annotations were conducted using hmmscan (HMMER) against the CAZy database. The functional contributions of microbial taxa at the species level were analyzed using custom R scripts. The relative contribution of each taxon to a functional pathway was calculated as its gene abundance divided by the total abundance of all taxa associated with that pathway. Values were normalized to sum to 1 per sample (26).

### Untargeted metabolomic analysis of rumen fluid

Rumen fluid samples (100 mL) were collected, and 100 μL aliquots were transferred into 1.5 mL centrifuge tubes. Each aliquot was mixed with 400 μL of extraction solution (acetonitrile-methanol, 1:1, vol/vol) containing 0.02 mg/mL L-2-chlorophenylalanine as the internal standard. Samples were vortexed for 30 s, followed by ultrasonication at 5°C and 40 kHz for 30 min. Protein precipitation was induced by incubating samples at −20°C for 30 min. The samples were then centrifuged at 13,000 × *g* for 15 min at 4°C. The resulting supernatants were collected and evaporated to dryness under a nitrogen stream. Dried samples were reconstituted in 100 μL of acetonitrile-water (1:1, vol/vol), sonicated for 5 min at 5°C (40 kHz), and centrifuged at 13,000 × *g* for 10 min at 4°C. The final supernatants were transferred to liquid chromatography-mass spectrometry (LC-MS) vials for analysis. Metabolomic profiling was performed on a Vanquish UHPLC system coupled with a Q Exactive HF Orbitrap mass spectrometer (both from Thermo Fisher Scientific, Waltham, MA, USA). Twelve samples met the quality criteria for untargeted metabolomic analysis and were included in downstream analyses.

Raw LC-MS data were processed using Progenesis QI (Waters Corporation, Milford, MA, USA) and analyzed via the Majorbio Cloud Platform. Metabolic features detected in at least 80% of samples in any group were retained. For features below the limit of quantification, values were imputed using the minimum value, and all data were normalized by sum. Variables with a relative standard deviation >30% in quality control samples were excluded. The remaining data were log_10_-transformed to generate the final data matrix for subsequent statistical analyses.

Multivariate analysis was performed on the processed matrix. Principal component analysis (PCA) and orthogonal partial least squares discriminant analysis (OPLS-DA) were conducted using the ropls R package. Model performance was assessed by sevenfold cross-validation and further validated using 200 permutation tests. All permuted *Q*² values were negative or near zero (*R*² = 0.806, *Q*² = −0.144), confirming the absence of overfitting (Fig. S1). Differential metabolites were identified using a combination of variable importance in projection (VIP > 1) from the OPLS-DA model and Student’s *t*-test (*P* < 0.05). No multiple testing correction was applied to metabolomic *t*-tests, as VIP filtering served as a feature selection step.

Differential metabolites were mapped to biochemical pathways using the KEGG database (http://www.genome.jp/kegg/). Pathway classification and enrichment analysis were conducted to determine the biological functions of the differential metabolites. Enrichment was performed using the “scipy.stats” module from Python (https://docs.scipy.org/doc/scipy/), identifying significantly enriched pathways related to the experimental treatments.

### Statistical analysis

The statistical analysis aimed to assess the effects of two dietary treatments on rumen VFAs and the rumen microbiome. For rumen VFA data meeting assumptions of normality and homogeneity of variance, an independent-samples *t*-test was performed using SPSS (version 27.0, IBM Corp., Armonk, NY, USA) to compare mean differences between groups.

Metagenomic microbiome and functional profile data were analyzed using the Majorbio Cloud Platform (https://cloud.majorbio.com) (27). Principal coordinate analysis (PCoA) and PCA, based on Bray-Curtis dissimilarity, were performed to visualize differences in community structure among samples. ADONIS with 999 permutations was then applied to test the statistical significance of overall community composition differences between groups. At the individual feature level, the Wilcoxon rank-sum test was used to identify differential abundance in taxonomic and functional features. Linear discriminant analysis effect size (LEfSe) was employed to detect significantly enriched KEGG pathways (LDA score > 2.0, *P* < 0.05). The underlying Kruskal-Wallis tests were FDR-corrected, and only features with *P* < 0.05 were reported as significant.

## RESULTS

### Rumen fermentation parameters

As shown in Table 2, the concentrations and molar proportions of propionate and butyrate were significantly higher in the FG group than in the HG group (*P* < 0.05). Conversely, the acetate molar proportion and acetate-to-propionate ratio were significantly higher in the HG group than in the FG group (*P* < 0.05). In contrast, no significant differences were observed in total VFA concentration or acetate concentration (*P* > 0.05).

**TABLE 2:** Comparison of rumen fermentation parameters between the two groups of Mongolian cattle^*a*^

Ruminal parameters	HG	FG	SEM	*P* values
Concentration (mmol/L)				
Acetate	49.49	47.80	0.817	0.065
Propionate	10.72^b^	12.67^a^	0.206	<0.001
Butyrate	7.72^b^	8.82^a^	0.268	0.002
Others VFAs	1.88^b^	2.75^a^	0.208	<0.001
Total VFA	69.81	72.03	1.017	0.054
Proportion (%)				
Acetate	70.88^a^	66.36^b^	0.468	<0.001
Propionate	15.36^b^	17.58^a^	0.177	<0.001
Butyrate	11.06^b^	12.24^a^	0.343	0.006
Others VFAs	2.70^b^	3.81^a^	0.296	0.004
Acetate:Propionate	4.62^b^	3.77^a^	0.064	<0.001

^*a*^ Different superscripts (^a,b^) within the same row denote significant differences (*P* < 0.05). Values are expressed as means ± SEM.

### Changes of rumen microbial communities

Rumen metagenomic sequencing generated 1,069,072,464 raw reads, with an average of 89,089,372 ± 5,799,900 reads per sample (mean ± SD). Following quality filtering and host genome removal, 1,050,475,550 high-quality reads were retained, averaging 87,539,629 ± 5,897,312 reads per sample. Contig assembly yielded 19,817,220 contigs, with a mean of 1,651,435 ± 238,969 contigs per sample (Table S1).

**Fig 1:**
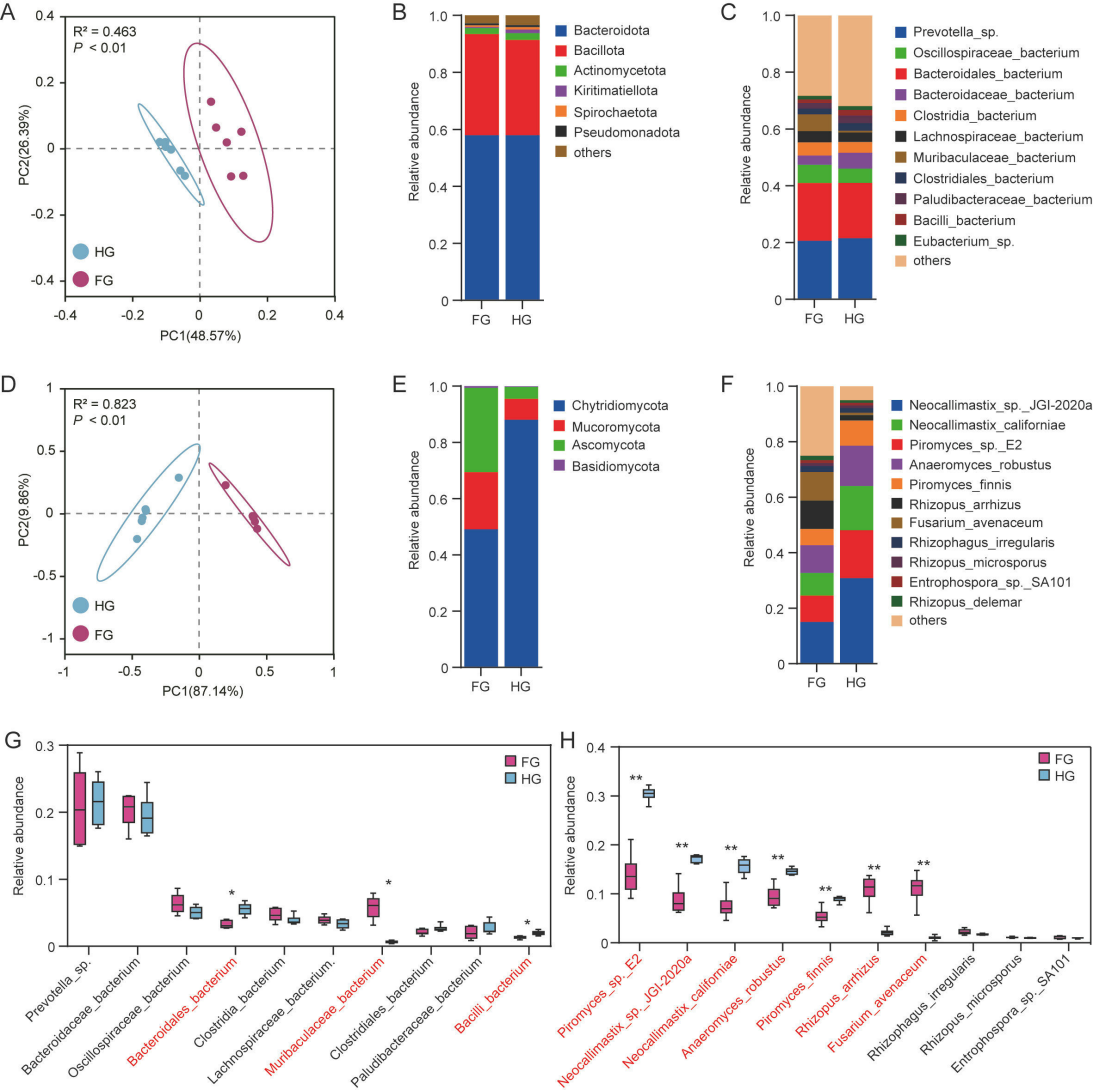
Composition and intergroup differences in ruminal bacterial and fungal communities of Mongolian cattle. (**A**) PCA of ruminal bacterial communities based on Bray-Curtis distances; group-level differences assessed using the ADONIS test. (**B**) Relative abundance of bacterial taxa at the phylum level. (**C**) Relative abundance of bacterial taxa at the species level. (**D**) PCA of ruminal fungal communities based on Bray-Curtis distances; group-level differences assessed using the ADONIS test. (**E**) Relative abundance of fungal taxa at the phylum level. (**F**) Relative abundance of fungal taxa at the species level. (**G**) Differential abundance of core bacterial species between the two groups. (**H**) Differential abundance of core fungal species between the two groups. FG, fresh grass-fed group; HG, hay-fed group. Error bars represent SD; statistical significance determined using the Wilcoxon rank-sum test (*P < 0.05, **P < 0.01).

The rumen metagenome of Mongolian cattle was predominantly composed of bacteria (91.03%), followed by archaea (5.72%), eukaryotes (2.21%), viruses (1.03%), and unclassified microorganisms (0.02%) (Table S2). The Wilcoxon rank-sum test revealed that bacterial and eukaryotic relative abundances were significantly higher in the HG group than in the FG group, whereas archaeal abundance was significantly lower (*P* < 0.05, Fig. S2). To elucidate the mechanisms underlying ruminal fiber degradation, we analyzed the composition and intergroup differences in bacterial and fungal communities. β-diversity analysis based on Bray-Curtis distances and visualized by PCA revealed distinct clustering and significant separation between the HG and FG groups for both bacterial and fungal communities, indicating divergent structures under different feeding regimes (Fig. 1A and D). At the phylum level, Bacteroidota (57.91%) and Bacillota (34.49%) dominated bacterial communities, with *Prevotella* sp. (21.08%) and Bacteroidales bacterium (19.87%) as the most abundant species (Fig. 1B and C). The dominant fungal phylum was Chytridiomycota (68.58%), Ascomycota (17.10%), and Mucoromycota (13.87%), with *Neocallimastix* sp. JGI-2020a (22.92%), *Piromyces* sp. E2 (13.37%), *Neocallimastix californiae* (12.24%), and *Anaeromyces robustus* (12.09%) as the major species (Fig. 1E and F). Among the top 10 bacterial species, *Bacteroidales bacterium* and *Bacilli bacterium* were significantly more abundant in the HG group than in the FG group, whereas *Muribaculaceae *bacterium was significantly more abundant in the FG group (*P* < 0.05, Fig. 1G). Among the top 10 fungal species, *Neocallimastix* sp. JGI-2020a, *Piromyces* sp. E2, *Neocallimastix californiae*, *Anaeromyces robustus*, and *Piromyces finnis* exhibited were significantly more abundant in the HG group, whereas *Rhizopus arrhizus* and *Fusarium avenaceum* were significantly more abundant in the FG group (*P* < 0.05, Fig. 1H).

### Cazyme profiles of the rumen microbial communities

**Fig 2:**
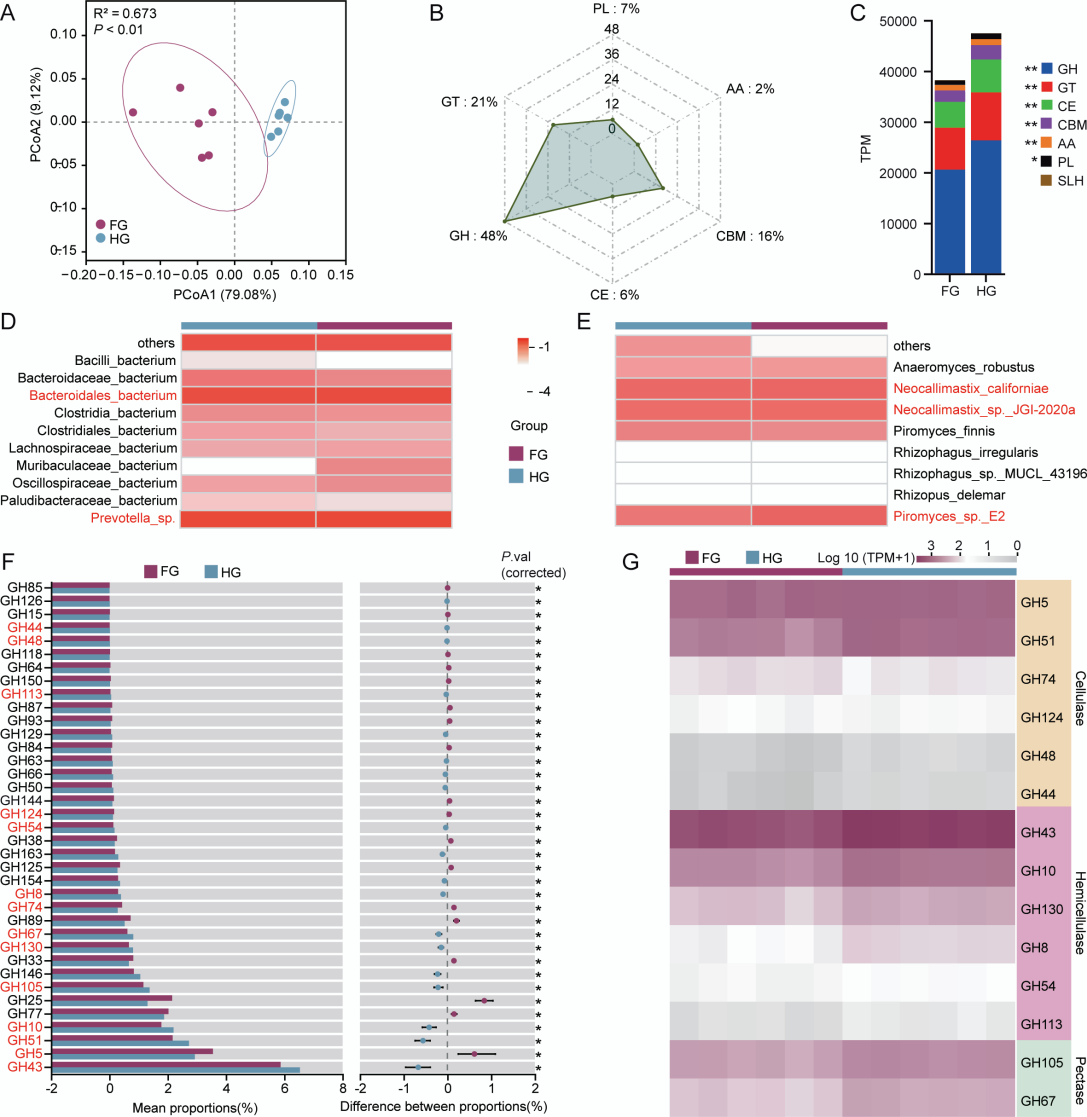
Composition and intergroup differences in CAZymes in the rumen of Mongolian cattle. (**A**) PCoA of ruminal CAZyme profiles based on Bray-Curtis distances; group-level differences assessed using the ADONIS test. (**B**) Relative proportion of CAZyme class. (**C**) Absolute abundance (TPM) of CAZyme class. (**D**) Functional contribution of the top 10 bacterial species to GH-class CAZymes. (**E**) Functional contribution of the top 10 fungal species to GH-class CAZymes. (**F**) GH families with significant differences in abundance between the two groups. (**G**) GH families involved in the degradation of cellulose, hemicellulose, and pectin among the differentially abundant GHs. FG, fresh grass-fed group; HG, hay-fed group. Error bars represent SD; statistical significance determined using the Wilcoxon rank-sum test (*P < 0.05, **P < 0.01).

β-diversity analysis based on Bray-Curtis distances and visualized via PCoA revealed significant differences in CAZyme profiles between the FG and HG groups (Fig. 2A). Among all identified CAZyme families, glycoside hydrolase (GH) families were the most abundant in both groups and showed significantly higher abundance in the HG group than in the FG group (*P* < 0.05, Fig. 2B and C). Functional contribution analysis showed that *Prevotella *sp. and Bacteroidales bacterium were the primary bacterial contributors to GHs, whereas *Neocallimastix *sp. JGI-2020a, *Piromyces *sp. E2, and *Neocallimastix californiae* were the dominant fungal contributors (Fig. 2D and E). Comparative analysis identified 37 GHs with significantly different abundances between groups (*P* < 0.05, Fig. 2F). In particular, the HG group exhibited elevated abundances of GH families associated with hemicellulose and pectin degradation. Specifically, GH43, GH10, GH130, GH8, GH54, and GH113 were linked to hemicellulose degradation, while GH105 and GH67 were associated with pectin degradation (Fig. 2G).

### Functional characteristics of the rumen microbial communities

**Fig 3:**
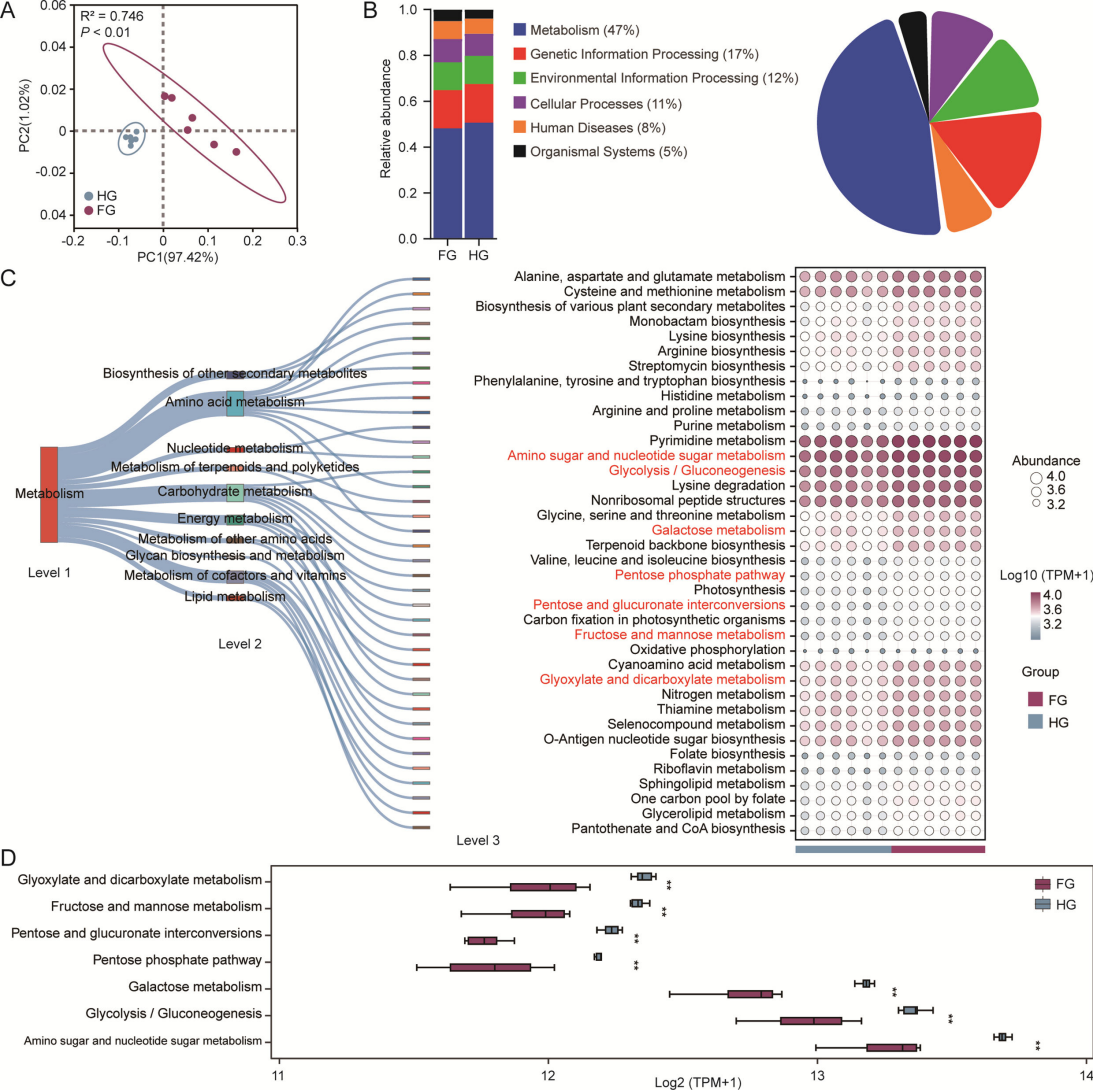
Functional characteristics of Mongolian cattle mediated by ruminal microorganisms. (**A**) PCA of ruminal functional profiles based on Bray-Curtis distances; group-level differences assessed using the ADONIS test. (**B**) Relative abundance and proportion of KEGG level 1 pathways. (**C**) Sankey diagram illustrating the distribution of metabolic pathways, and a bubble diagram based on KEGG level 3 pathways. (**D**) Box plots of differential KEGG level 3 pathways within the carbohydrate metabolism category. FG, fresh grass-fed group; HG, hay-fed group. Error bars represent SD; statistical significance determined using the Wilcoxon rank-sum test (*P < 0.05, **P < 0.01).

β-diversity analysis based on Bray-Curtis distances and visualized using PCA revealed significant differences in rumen microbial functional profiles between the FG and HG groups (Fig. 3A). According to KEGG level 1 functional classification, metabolism was the predominant category, highlighting its central role in rumen microbial activity (Fig. 3B). To further elucidate metabolic differences, LEfSe was applied to KEGG level 3 functions within the metabolism category, identifying 38 significantly enriched pathways (LDA score > 2.0, *P* < 0.05). These enriched pathways were primarily associated with carbohydrate and amino acid metabolism. Given the study’s focus on ruminal fiber degradation mechanisms, particular emphasis was placed on carbohydrate metabolism pathways (Fig. 3C). Seven KEGG level 3 pathways showed significant differences between groups, all enriched in the HG group (*P* < 0.05). These included amino sugar and nucleotide sugar metabolism, glycolysis/gluconeogenesis, galactose metabolism, the pentose phosphate pathway, pentose and glucuronate interconversions, fructose and mannose metabolism, and glyoxylate and dicarboxylate metabolism (Fig. 3D).

### Rumen metabolome analysis

**Fig 4:**
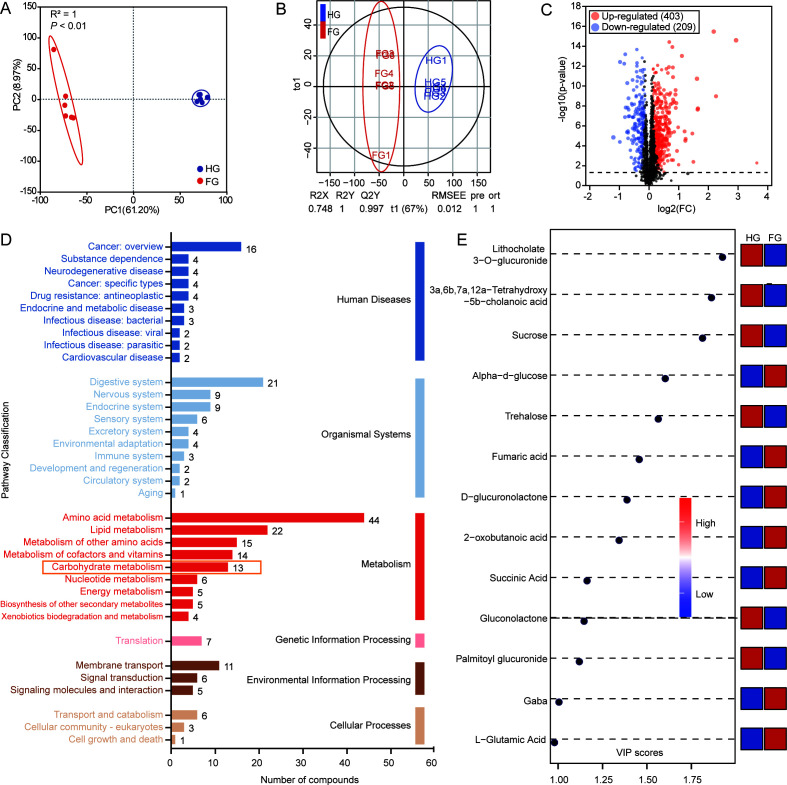
Analysis of the rumen metabolome in Mongolian cattle. (**A**) PCA of ruminal metabolites. (**B**) OPLS-DA of ruminal metabolites. (**C**) Volcano plot of differential metabolites. (**D**) Pathway classification of all identified differential metabolites. (**E**) Differential metabolites involved in carbohydrate metabolism. FG, fresh grass-fed group; HG, hay-fed group.

To further elucidate the characteristics of ruminal metabolites and their associations with fiber-degrading bacteria, untargeted LC-MS metabolomic analysis was conducted on 12 rumen fluid samples. PCA revealed distinct separation of ruminal metabolite profiles between the HG and FG groups, indicating significant differences in their metabolomes (Fig. 4A). OPLS-DA further confirmed this separation with robust predictive performance (R^²^Y = 0.748, Q^²^Y = 0.997) (Fig. 4B). Volcano plot analysis identified 612 differential metabolites between groups, with 403 upregulated and 209 downregulated in HG versus FG (Fig. 4C). These 612 differential metabolites were annotated using the KEGG database, mapping to 36 KEGG pathways. Pathways containing more than 10 compounds included those related to cancer, digestive system, amino acid metabolism, lipid metabolism, metabolism of other amino acids, metabolism of cofactors and vitamins, carbohydrate metabolism, and membrane transport (Fig. 4D). To investigate the mechanisms underlying ruminal fiber degradation, we focused on 13 metabolites associated with the carbohydrate metabolism pathway. Six metabolites were enriched in the HG group: lithocholate 3-O-glucuronide, 3α,6β,7α,12α-tetrahydroxy-5β-cholanoic acid, sucrose, trehalose, gluconolactone, and palmitoyl glucuronide. Conversely, seven metabolites were enriched in the FG group: α-d-glucose, fumaric acid, d-glucuronolactone, 2-oxobutanoic acid, succinic acid, γ-aminobutyric acid (GABA), and l-glutamic acid (Fig. 4E).

**Fig 5:**
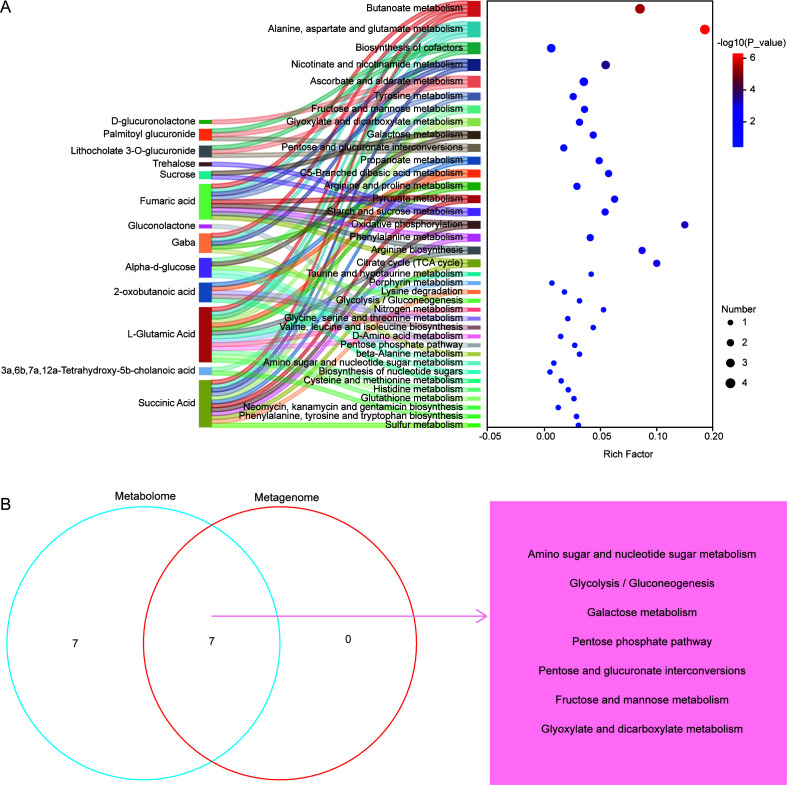
Analysis of the rumen metabolome in Mongolian cattle. (**A**) Pathway enrichment analysis of 13 differential metabolites. (**B**) Common pathways identified between metagenomic and metabolomic analyses.

KEGG enrichment analysis of the 13 differential metabolites in carbohydrate metabolism identified 14 enriched pathways: amino sugar and nucleotide sugar metabolism, pentose phosphate pathway, glycolysis/gluconeogenesis, citrate cycle (TCA cycle), starch and sucrose metabolism, pyruvate metabolism, C5-branched dibasic acid metabolism, propanoate metabolism, pentose and glucuronate interconversions, galactose metabolism, glyoxylate and dicarboxylate metabolism, fructose and mannose metabolism, ascorbate and aldarate metabolism, and butanoate metabolism (Fig. 5A). Of these, seven pathways overlapped between metabolomics and metagenomics analyses: amino sugar and nucleotide sugar metabolism, glycolysis/gluconeogenesis, galactose metabolism, pentose phosphate pathway, pentose and glucuronate interconversions, fructose and mannose metabolism, and glyoxylate and dicarboxylate metabolism (Fig. 5B).

### Integrated analysis of omics and phenotype associations

**Fig 6:**
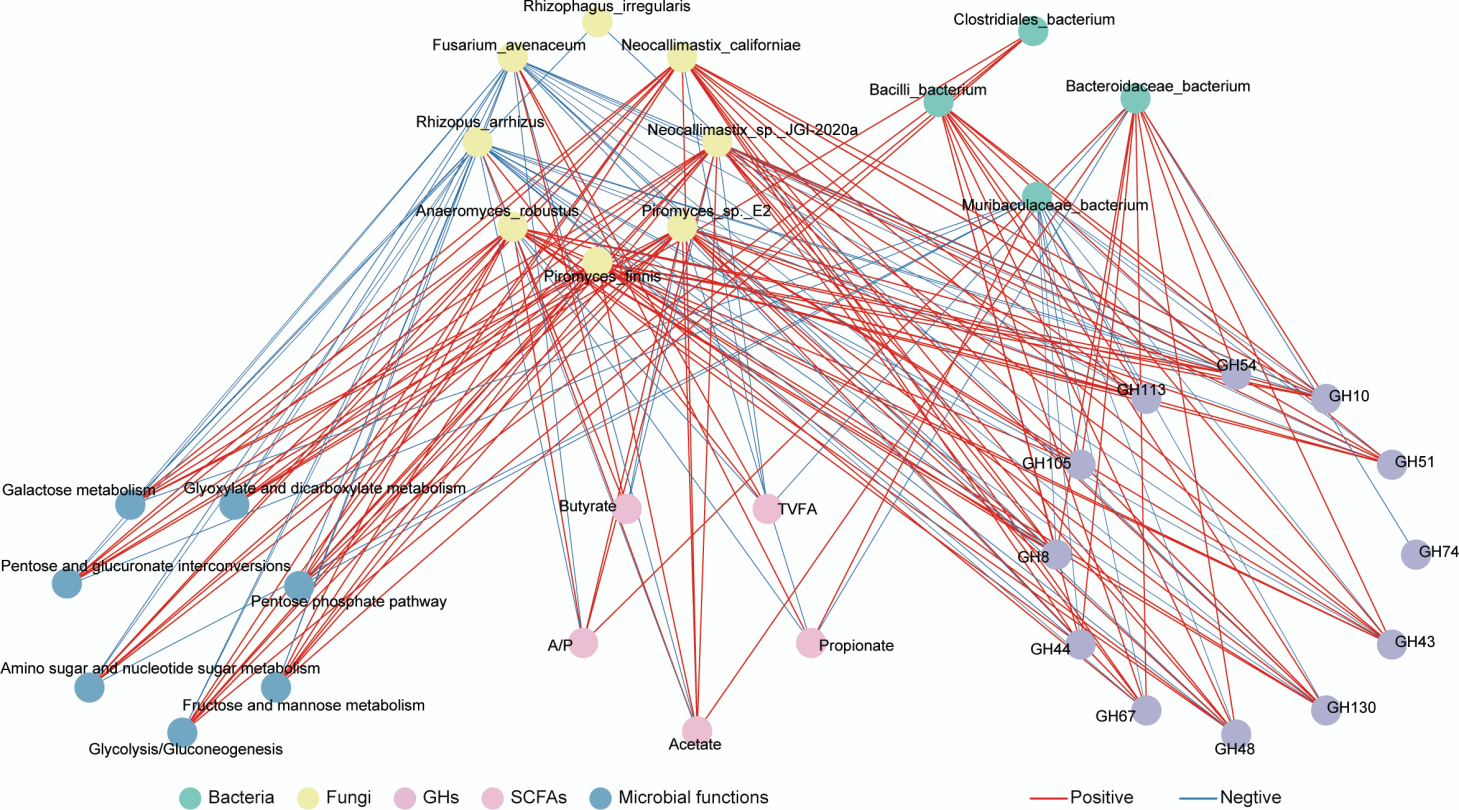
Correlation analysis of bacterial and fungal communities, microbial functions, GHs, VFA, and DEMs.

Spearman’s rank correlation analysis was performed to evaluate relationships among the top 10 differential microbial species, GH families, microbial functional pathways, and VFAs. Only correlations with |*r*| > 0.7 and *P* < 0.05 were retained for interpretation (Fig. 6). Bacteroidaceae bacterium showed significant positive correlations with acetate and GH families linked to hemicellulose and pectin degradation. Muribaculaceae bacterium showed a significant positive correlation with propionate. Among fungal species, *Anaeromyces robustus*, *Neocallimastix* sp. JGI-2020a, *Piromyces* sp. E2, *Neocallimastix californiae*, and *Piromyces finnis* showed strong positive correlations with acetate, pathways of glycolysis/gluconeogenesis, the pentose phosphate pathway, and fructose and mannose metabolism, and GH families linked to hemicellulose degradation.

## DISCUSSION

VFAs contribute approximately 70–80% of the metabolizable energy in ruminants, with their synthesis and proportions strongly influenced by dietary nutrient composition (28). In this study, the molar proportion of propionate was significantly higher in the FG group than in the HG group, whereas that of acetate was significantly lower. Consequently, the acetate-to-propionate ratio was significantly reduced in the FG group. This shift may be attributed to the higher fiber content in the HG diet. Notably, diets high in NDF and ADF stimulate rumination and salivary secretion; bicarbonate and phosphate in saliva act as physiological buffers, stabilizing ruminal pH and creating an environment that favors acetate-producing microbial populations (29). Previous studies have demonstrated that structural carbohydrates, primarily cellulose and hemicellulose, promote acetate production during ruminal fermentation, whereas diets rich in non-structural carbohydrates, such as starch, enhance propionate synthesis (17, 30). The observed shift in acetate molar proportion in this study aligns closely with these findings. Furthermore, Zhao et al. (31) reported a similar acetate-dominated fermentation pattern in yak cows fed low-quality roughage, supporting the idea that hay-based diets shift rumen fermentation toward acetate production in ruminants. Collectively, these results confirm that Mongolian cattle exhibit an acetate-dominated rumen fermentation profile under low-quality hay-based diets.

In the metagenomic analysis, bacteria were the predominant microbial domain in the rumen microbiota of Mongolian cattle, consistent with prior ruminant studies and reflecting their critical role in degrading plant structural carbohydrates and producing fermentation products (16). Notably, ruminal fungi, characterized by their highly branched rhizoidal structures and invasive growth, can penetrate plant cell wall regions inaccessible to bacteria, facilitating deeper degradation of complex polysaccharides in synergy with bacteria (32). Comparative analysis of bacterial and fungal communities between the FG and HG groups revealed multiple microbial taxa with key roles in fiber degradation. At the bacterial species level, Bacteroidales bacterium showed significantly higher relative abundance in the HG group, likely due to the elevated NDF and ADF content in the hay-based diet. Pu et al. (33) previously reported that increasing dietary fiber from 16.7% to 24.1% significantly increased Bacteroidales bacterium abundance in the pig cecum, highlighting its adaptability to high-fiber diets. Similarly, Zhang et al. (34) demonstrated that Bacteroidales bacterium expresses a diverse array of CAZymes capable of efficiently degrading structural polysaccharides (e.g., cellulose, hemicellulose, and pectin) and producing VFAs, including acetate, propionate, and butyrate. The significant enrichment of Bacteroidales bacterium in the HG group suggests its pivotal role in acetate production and host energy homeostasis, partly explaining the increased acetate levels observed in this group. However, the high structural complexity of cellulose-lignin cross-links in hay limits bacterial degradation capacity alone, necessitating synergistic interactions with fungi for effective lignocellulose breakdown. At the fungal species level, *Neocallimastix* sp. JGI-2020a, *Piromyces* sp. E2, *Neocallimastix californiae*, and *Anaeromyces robustus* showed significantly higher relative abundances in the HG group. These fungi are known for efficiently degrading lignified plant fibers, with their abundance positively correlated with dietary NDF content. Their fermentation products include formate, acetate, and lactate (35–38). The increased abundance of these lignocellulose-degrading fungi in the HG group is likely attributable to the higher lignocellulose content in the hay-based diet, which stimulated their growth and enzymatic activity. This finding aligns with Liang et al. (36), who reported enhanced fungal activity during *in vitro* rumen fermentation of corn stover in Angus cattle. Collectively, these results highlight a synergistic microbial strategy wherein fiber-adapted bacteria and lignocellulolytic fungi collaborate to overcome the structural complexity of hay, enhancing fiber utilization efficiency and supporting host energy metabolism under low-nutrient conditions.

CAZymes are critical enzymes secreted by ruminal microorganisms that catalyze the degradation of plant cell wall polysaccharides into fermentable monosaccharides, such as hexoses (e.g., glucose) and pentoses (e.g., xylose), which serve as key precursors for VFA production (39, 40). In this study, GH families were the most abundant CAZyme category in both the FG and HG groups. These enzymes hydrolyze glycosidic bonds in complex carbohydrates, facilitating the cleavage of polysaccharide chains and the release of fermentable sugars (41, 42). The GH-dominated CAZyme profile observed here aligns with findings by Li et al. (43) in the rumen microbiota of goats, suggesting a conserved fiber degradation strategy across ruminants. Functional contribution analysis identified *Prevotella* sp. and Bacteroidales bacterium as the major bacterial producers of GHs, and *Neocallimastix* sp. JGI-2020a, *Piromyces* sp. E2, and *Neocallimastix californiae* as the primary fungal contributors. Previous studies indicate that *Prevotella* contributes approximately 30% of hemicellulase-encoding genes, particularly those involved in xylan degradation (e.g., GH 10, GH 43, and GH 8) (44). Similarly, Bacteroidales bacterium encodes cellulases from GH 5, GH 51, and GH 124 families, enabling the breakdown of cellulose-rich substrates (45). Although functional annotations of fungal CAZymes are limited, available data suggest that *Neocallimastix* and *Piromyces* species express GH 11, GH 43, and GH 48, particularly under high-lignocellulose conditions (46). In this study, GH families associated with hemicellulose and pectin degradation were significantly enriched in the HG group, indicating enhanced breakdown of matrix components surrounding plant cell walls, which likely improves microbial access to the cellulose core. This enzymatic adaptation may underpin the rumen microbiota’s enhanced capacity to extract nutrients from fibrous, less digestible diets. The increased relative abundance of microbial taxa contributing to these GHs in the HG group further supports this interpretation, reflecting an adaptive shift in the microbial community toward enhanced GH-mediated polysaccharide degradation under hay-based feeding conditions. However, direct measurement of hemicellulase and pectinase activities was not performed in this study. Future research integrating enzyme activity assays with multi-omics approaches will further validate the functional roles of these GHs in fiber degradation and enhance mechanistic understanding of their contributions to ruminal polysaccharide breakdown.

Metagenomic KEGG pathway analysis revealed seven significantly enriched carbohydrate metabolism pathways in the HG group: glycolysis/gluconeogenesis, galactose metabolism, pentose phosphate pathway, pentose and glucuronate interconversions, fructose and mannose metabolism, glyoxylate and dicarboxylate metabolism, and amino sugar and nucleotide sugar metabolism. Notably, pathways enriched with differential metabolites in untargeted metabolomics were consistent with those identified in metagenomics. These pathways metabolize monosaccharides derived from fiber degradation into pyruvate and VFAs, indicating that a high-fiber hay diet enhances the metabolic activity and fermentation efficiency of fiber-degrading microbes in the rumen of Mongolian cattle. Similar findings were reported by Zheng et al. (47), who observed upregulated glycolysis/gluconeogenesis and lipid metabolism pathways in grazing yaks on the Qinghai-Tibetan Plateau, particularly during periods of forage scarcity, contributing to energy homeostasis. Likewise, Jiang et al. (48) reported enhanced KEGG functions related to carbohydrate degradation in the rumen microbiome of grazing Mongolian cattle than in housed cattle. These comparisons underscore that high-fiber diets consistently enhance rumen carbohydrate metabolism, although specific pathways and microbial responses may vary depending on feed type and environmental conditions. This study found significantly increased levels of fumaric acid and succinic acid in the rumen of Mongolian cattle fed FG. These metabolites are key intermediates in the succinate-propionate pathway, suggesting that FG intake promotes rumen propionate fermentation, consistent with rumen fermentation characteristics (49). The high carbohydrate content in FG is hydrolyzed by microbes into greater amounts of glucose, which is subsequently metabolized via glycolysis and the succinate pathway to produce propionate. In the HG group, gluconolactone accumulation serves as a key metabolic adaptation that enhances acetate production under high-fiber conditions via two interconnected mechanisms. Gluconolactone, a lactone derivative of gluconic acid, is rapidly hydrolyzed by gluconolactonase to gluconic acid (50). The latter either enters the Entner-Doudoroff pathway or is phosphorylated to 6-phosphogluconate (50). These pathways channel carbon to pyruvate and acetyl-CoA, which acetogens convert to acetate via the Wood-Ljungdahl pathway (51). More importantly, this metabolic flux precisely regulates ruminal redox balance. In the oxidative pentose phosphate pathway, 6-phosphogluconate is decarboxylated by 6-phosphogluconate dehydrogenase to ribulose-5-phosphate, generating 2 mol NADPH per glucose equivalent (52). NADPH, on one hand, reduces oxidized ferredoxin, providing the necessary reducing power for the conversion of pyruvate to acetyl-CoA, thereby promoting acetate production; on the other hand, it provides reducing equivalents to mitigate oxidative stress from lignin-derived phenolics, protecting oxygen-sensitive acetogens (51, 53). Thus, gluconolactone accumulation supplies carbon while sustaining a reducing environment, thereby promoting efficient acetate production in the slow-fermenting rumen.

Furthermore, a phenotype-omics association network highlighted key microbial contributors to GH production and VFA synthesis. In the HG group, Bacteroidaceae bacterium, along with *Anaeromyces robustus*, *Neocallimastix* sp. JGI-2020a, *Piromyces* sp. E2, *Neocallimastix californiae*, and *Piromyces finnis* exhibited significant positive correlations with GH families involved in hemicellulose degradation, VFA-producing metabolic pathways, and acetate production. These associations align with their increased relative abundances, suggesting that Mongolian cattle rely on a specialized consortium of fiber-adapted bacteria and fungi to degrade complex plant polymers. Collectively, these findings reveal a finely tuned rumen microbial ecosystem that enables efficient fiber utilization and acetate-dominated fermentation, reflecting an adaptive energy strategy in Mongolian cattle fed low-quality forage. It is important to note that the study design prioritized rumen function under maintained body condition to elucidate adaptive mechanisms relevant to extensive grazing systems, rather than whole-animal performance metrics. Consequently, growth performance was not evaluated, primarily due to the inherent challenges of monitoring individual feed intake and weight gain in a free-grazing system. This focused approach provides foundational insights into microbial-driven energy efficiency, paving the way for future studies integrating performance tracking with multi-omics to fully link rumen adaptations to host productivity.

### Conclusion

Compared with FG, which is relatively nutrient-rich and highly digestible, a high-fiber hay diet in Mongolian cattle induces a microbial shift toward greater relative abundance of fiber-degrading microorganisms, including Bacteroidales bacteria and *Neocallimastix* spp. This shift may promote the production of enzymes involved in hemicellulose degradation (GH 43 and GH 10) and pectin degradation (GH 105). Furthermore, the upregulation of metabolites, including gluconolactone, indirectly promotes acetate production through pathways such as glycolysis/gluconeogenesis and the pentose phosphate pathway. These findings highlight the adaptive microbial and metabolic responses of Mongolian cattle to a high-fiber forage diet.

## Data Availability

The metagenomic sequencing raw data for our samples have been deposited in the NCBI Sequence Read Archive (SRA) under accession number PRJNA1321689.

## SUPPLEMENTAL MATERIAL

Figures S1 and S2; Tables S1 to S6 spectrum.03051-25-s0001.docx Fig. S1: Permutation test confirming no overfitting. Fig. S2: Analysis of domain-level differences in rumen microorganisms of mongolian cattle. Table S1: The quality of metagenome. Table S2: Analysis of domain-level differences in rumen microorganisms of Mongolian cattle. Table S3: Intergroup differences in the ruminal bacterial communities of mongolian cattle. Table S4: Intergroup differences in the ruminal fungal communities of mongolian cattle. Table S5: Differential analysis of gh family in CAZymes. Table S6: Differential analysis of carbohydrate metabolism pathways in KEGG level 3.

ASM does not own the copyrights to Supplemental Material that may be linked to, or accessed through, an article. The authors have granted ASM a non-exclusive, world-wide license to publish the Supplemental Material files. Please contact the corresponding author directly for reuse.
