# Supplementary material for: Metagenomic and metabolomic analyses of rumen fiber digestion in Mongolian cattle fed fresh grass versus hay
Source: Microbiol Spectr. 2026 Jan 8;14(2):e03051-25. doi: 10.1128/spectrum.03051-25 (PMC12889088; doi:10.1128/spectrum.03051-25)
Supplement: Figures S1 and S2; Tables S1 to S6 — Fig. S1: Permutation test confirming no overfitting. Fig. S2: Analysis of domain-level differences in rumen microorganisms of mongolian cattle. Table S1: The quality of metagenome. Table S2: Analysis of domain-level differences in rumen microorganisms of Mongolian cattle. Table S3: Intergroup differences in the ruminal bacterial communities of mongolian cattle. Table S4: Intergroup differences in the ruminal fungal communities of mongolian cattle. Table S5: Differential analysis of gh family in CAZymes. Table S6: Differential analysis of carbohydrate metabolism pathways in KEGG level 3. [file spectrum.03051-25-s0001.docx]

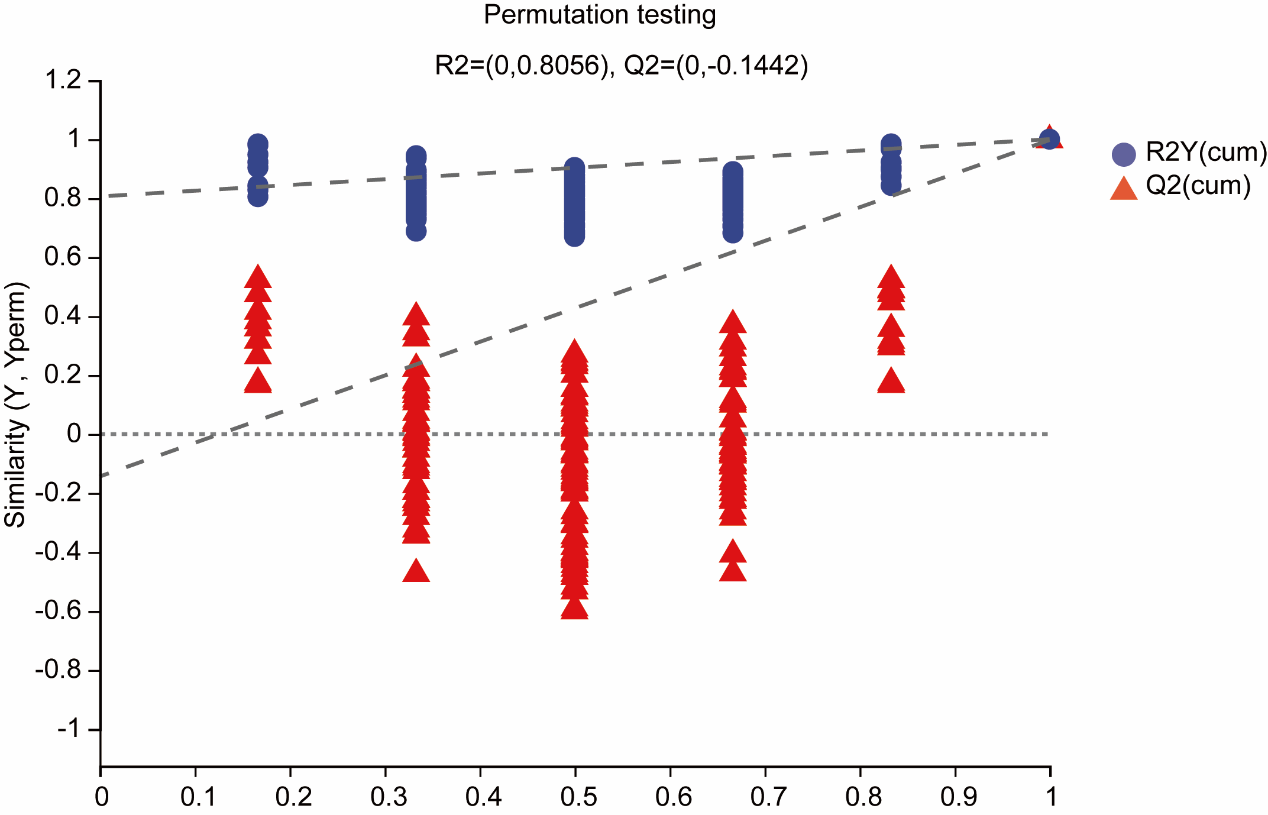


**Fig. S1** Permutation test confirming no overfitting. All 200 permuted Q² values were negative or near zero (R² = 0.806, Q² = –0.144).

Table S1 The quality of metagenome

| Samples | Raw reads | Clean reads  after QC | Contigs | N50(bp) | ORFs |
| --- | --- | --- | --- | --- | --- |
| FG1 | 86,384,556 | 85,121,000 | 1,764,747 | 586 | 2,091,047 |
| FG2 | 87,799,660 | 85,972,886 | 1,638,074 | 569 | 1,918,719 |
| FG3 | 86,968,170 | 85,413,424 | 1,875,368 | 543 | 2,176,558 |
| FG4 | 86,996,268 | 85,522,496 | 1,758,104 | 541 | 2,109,179 |
| FG5 | 99,204,722 | 98,459,246 | 2,195,283 | 573 | 2,540,232 |
| FG6 | 86,085,002 | 84,918,672 | 1,712,383 | 538 | 1,964,853 |
| HG1 | 102,046,864 | 100,639,794 | 1,678,168 | 609 | 2,095,444 |
| HG2 | 86,761,290 | 85,105,820 | 1,485,139 | 587 | 1,816,067 |
| HG3 | 86,536,062 | 84,924,648 | 1,384,869 | 594 | 1,708,603 |
| HG4 | 88,273,046 | 85,972,376 | 1,522,527 | 556 | 1,843,458 |
| HG5 | 90,554,498 | 88,123,428 | 1,481,721 | 559 | 1,788,608 |
| HG6 | 81,462,326 | 80,301,760 | 1,320,837 | 572 | 1,607,417 |
| Total | 1,069,072,464 | 1,050,475,550 | 19,817,220 | 6,827 | 23,660,185 |
| mean | 89,089,372.00 | 87,539,629.17 | 1,651,435.00 | 568.92 | 1,971,682.08 |
| SD | 5,799,900.34 | 5,897,312.18 | 238,969.24 | 22.47 | 250,928.91 |

ORF=open reading frame. HG, hay-fed group; FG, fresh grass-fed group.

Table S2 Analysis of domain-level differences in rumen microorganisms of Mongolian cattle

| Name | FG-Mean(%) | FG-Sd(%) | HG-Mean(%) | HG-Sd(%) | P-value | Corrected  p-value |
| --- | --- | --- | --- | --- | --- | --- |
| Bacteria | 86.828 | 3.747 | 95.220 | 0.545 | 0.005 | 0.008 |
| Archaea | 7.896 | 2.097 | 3.546 | 0.682 | 0.005 | 0.008 |
| Eukaryota | 0.308 | 0.154 | 4.105 | 1.666 | 0.005 | 0.008 |
| Viruses | 1.148 | 0.510 | 0.905 | 0.173 | 0.471 | 0.471 |
| unclassified | 0.022 | 0.002 | 0.021 | 0.002 | 0.174 | 0.217 |

HG, hay-fed group; FG, fresh grass-fed group. Error bars represent Sd; statistical significance determined by Wilcoxon rank-sum test (**P* < 0.05, ***P* < 0.01, ****P* < 0.001).


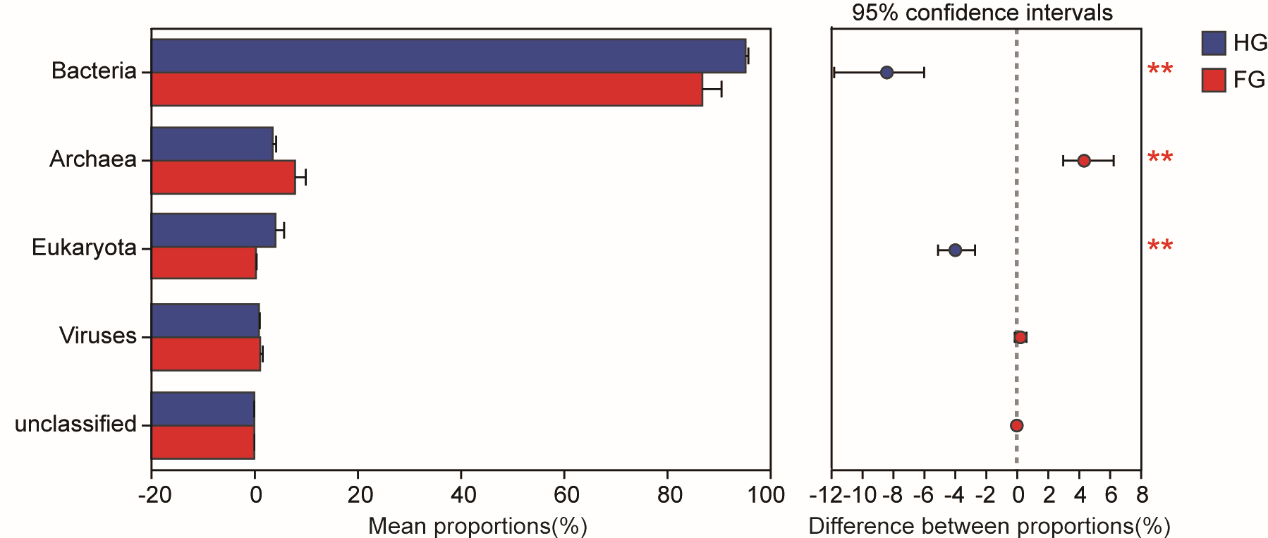


**Fig. S2** Analysis of domain-level differences in rumen microorganisms of mongolian cattle. HG, hay-fed group; FG, fresh grass-fed group. Error bars represent Sd; statistical significance determined by Wilcoxon rank-sum test (**P* < 0.05, ***P* < 0.01, ****P* < 0.001).

Table S3 Intergroup differences in the ruminal bacterial communities of mongolian cattle

| Name | FG-Mean(%) | FG-Sd(%) | HG-Mean(%) | HG-Sd(%) | P-value | Corrected P-value |
| --- | --- | --- | --- | --- | --- | --- |
|  |  |  |  |  |  |  |
| Prevotella_sp. | 20.79 | 5.502 | 21.53 | 3.275 | 0.81 | 0.81 |
| Bacteroidales_bacterium | 20.3 | 2.477 | 19.44 | 2.888 | 0.575 | 0.628 |
| Oscillospiraceae_bacterium | 6.36 | 1.461 | 5.041 | 0.8609 | 0.093 | 0.223 |
| Bacteroidaceae_bacterium | 3.253 | 0.6016 | 5.55 | 0.8836 | 0.005 | 0.03 |
| Clostridia_bacterium | 4.67 | 0.9974 | 3.859 | 0.7034 | 0.174 | 0.26 |
| Lachnospiraceae_bacterium | 3.925 | 0.5862 | 3.323 | 0.6901 | 0.174 | 0.26 |
| Muribaculaceae_bacterium | 5.813 | 1.691 | 0.6216 | 0.1899 | 0.005 | 0.03 |
| Clostridiales_bacterium | 2.083 | 0.4429 | 2.652 | 0.5076 | 0.128 | 0.256 |
| Paludibacteraceae_bacterium | 1.995 | 0.9182 | 2.69 | 0.9631 | 0.23 | 0.306 |
| Bacilli_bacterium | 1.282 | 0.2183 | 1.983 | 0.3313 | 0.008 | 0.033 |

HG, hay-fed group; FG, fresh grass-fed group. Error bars represent Sd; statistical significance determined by Wilcoxon rank-sum test (**P* < 0.05, ***P* < 0.01, ****P* < 0.001).

Table S4 Intergroup differences in the ruminal fungal communities of mongolian cattle

| Name | FG-Mean(%) | FG-Sd(%) | HG-Mean(%) | HG-Sd(%) | P-value | Corrected P-value |
| --- | --- | --- | --- | --- | --- | --- |
|  |  |  |  |  |  |  |
| Neocallimastix_sp.  _JGI-2020a | 0.139 | 0.041 | 0.304 | 0.015 | 0.005 | 0.008 |
| Piromyces_sp._E2 | 0.087 | 0.029 | 0.172 | 0.008 | 0.005 | 0.008 |
| Anaeromyces_robustus | 0.094 | 0.021 | 0.146 | 0.007 | 0.005 | 0.008 |
| Neocallimastix_californiae | 0.075 | 0.026 | 0.157 | 0.016 | 0.005 | 0.008 |
| Piromyces_finnis | 0.054 | 0.016 | 0.089 | 0.006 | 0.008 | 0.011 |
| Rhizopus_arrhizus | 0.110 | 0.027 | 0.021 | 0.007 | 0.005 | 0.008 |
| Fusarium_avenaceum | 0.111 | 0.030 | 0.010 | 0.004 | 0.005 | 0.008 |
| Rhizophagus_irregularis | 0.022 | 0.006 | 0.017 | 0.002 | 0.128 | 0.154 |
| Rhizopus_delemar | 0.011 | 0.002 | 0.009 | 0.002 | 0.105 | 0.108 |
| Entrophospora_sp._SA101 | 0.012 | 0.003 | 0.009 | 0.001 | 0.298 | 0.325 |

HG, hay-fed group; FG, fresh grass-fed group. Error bars represent Sd; statistical significance determined by Wilcoxon rank-sum test (**P* < 0.05, ***P* < 0.01, ****P* < 0.001).

Table S5 Differential analysis of gh family in CAZymes

| Name | FG-Mean(%) | FG-Sd(%) | HG-Mean(%) | HG-Sd(%) | P-value | Corrected  P-value |
| --- | --- | --- | --- | --- | --- | --- |
| GH43 | 5.871 | 0.300 | 6.538 | 0.238 | 0.008 | 0.035 |
| GH5 | 3.551 | 0.601 | 2.933 | 0.044 | 0.008 | 0.035 |
| GH10 | 1.783 | 0.130 | 2.200 | 0.155 | 0.005 | 0.026 |
| GH77 | 2.028 | 0.047 | 1.876 | 0.071 | 0.005 | 0.026 |
| GH105 | 1.166 | 0.109 | 1.380 | 0.076 | 0.008 | 0.035 |
| GH146 | 0.838 | 0.065 | 1.061 | 0.066 | 0.005 | 0.026 |
| GH130 | 0.671 | 0.064 | 0.813 | 0.042 | 0.005 | 0.026 |
| GH74 | 0.435 | 0.041 | 0.285 | 0.029 | 0.005 | 0.026 |
| GH8 | 0.297 | 0.033 | 0.396 | 0.032 | 0.005 | 0.026 |
| GH154 | 0.299 | 0.022 | 0.367 | 0.035 | 0.013 | 0.042 |
| GH125 | 0.364 | 0.023 | 0.278 | 0.036 | 0.005 | 0.026 |
| GH163 | 0.193 | 0.015 | 0.307 | 0.057 | 0.005 | 0.026 |
| GH38 | 0.263 | 0.032 | 0.184 | 0.018 | 0.005 | 0.026 |
| GH54 | 0.138 | 0.020 | 0.175 | 0.026 | 0.013 | 0.042 |
| GH124 | 0.164 | 0.031 | 0.123 | 0.016 | 0.013 | 0.042 |
| GH50 | 0.085 | 0.011 | 0.134 | 0.039 | 0.013 | 0.042 |
| GH63 | 0.094 | 0.011 | 0.115 | 0.012 | 0.013 | 0.042 |
| GH84 | 0.098 | 0.021 | 0.057 | 0.003 | 0.005 | 0.026 |
| GH93 | 0.098 | 0.023 | 0.046 | 0.008 | 0.005 | 0.026 |
| GH87 | 0.098 | 0.026 | 0.038 | 0.005 | 0.005 | 0.026 |
| GH113 | 0.036 | 0.009 | 0.062 | 0.018 | 0.008 | 0.035 |
| GH64 | 0.038 | 0.009 | 0.006 | 0.004 | 0.005 | 0.026 |
| GH118 | 0.021 | 0.008 | 0.008 | 0.002 | 0.013 | 0.042 |
| GH48 | 0.010 | 0.003 | 0.018 | 0.004 | 0.013 | 0.042 |
| GH15 | 0.017 | 0.009 | 0.006 | 0.002 | 0.008 | 0.035 |
| GH126 | 0.005 | 0.001 | 0.013 | 0.004 | 0.005 | 0.026 |

HG, hay-fed group; FG, fresh grass-fed group. Error bars represent Sd; statistical significance determined by Wilcoxon rank-sum test (**P* < 0.05, ***P* < 0.01, ****P* < 0.001).

Table S6 Differential analysis of carbohydrate metabolism pathways in KEGG level 3

| Name | FG-Mean(%) | FG-Sd(%) | HG-Mean(%) | HG-Sd(%) | P-value | Corrected P-value |
| --- | --- | --- | --- | --- | --- | --- |
|  |  |  |  |  |  |  |
| Amino sugar and nucleotide sugar metabolism | 13.690 | 0.023 | 13.260 | 0.155 | 0.005 | 0.005 |
| Glycolysis / Gluconeogenesis | 13.360 | 0.047 | 12.960 | 0.176 | 0.005 | 0.005 |
| Galactose metabolism | 13.180 | 0.025 | 12.730 | 0.159 | 0.005 | 0.005 |
| Glyoxylate and dicarboxylate metabolism | 12.350 | 0.036 | 11.960 | 0.200 | 0.005 | 0.005 |
| Fructose and mannose metabolism | 12.330 | 0.028 | 11.940 | 0.159 | 0.005 | 0.005 |
| Pentose phosphate pathway | 12.200 | 0.044 | 11.780 | 0.204 | 0.005 | 0.005 |
| Pentose and glucuronate interconversions | 12.230 | 0.035 | 11.740 | 0.121 | 0.005 | 0.005 |

HG, hay-fed group; FG, fresh grass-fed group. Error bars represent Sd; statistical significance determined by Wilcoxon rank-sum test (**P* < 0.05, ***P* < 0.01, ****P* < 0.001).
